# Positive parenting style and academic burnout in physical education teacher education students: a chain mediating model of physical activity and meaning in life

**DOI:** 10.3389/fpsyg.2026.1854548

**Published:** 2026-06-01

**Authors:** Hui Ma, Lijun Liu, Shuting Huang, Zhonggen Yin, Tong Liu

**Affiliations:** 1 School of Marxism, Chengdu Sport University, Chengdu, Sichuan, China; 2 Sichuan Provincial Sports Museum, Chengdu, Sichuan, China; 3 Chongqing Yucai Secondary School, Chongqing, China; 4 College of Physical Education and Health Management, Chongqing University of Education, Chongqing, China; 5 School of Sports Training, Chengdu Sport University, Chengdu, Sichuan, China

**Keywords:** academic burnout, meaning in life (MiL), physical activity, physical education teacher education students, positive parenting style

## Abstract

**Objective:**

Academic burnout impairs university students’ development, yet research on Physical Education Teacher Education (PETE) students facing both academic and high-intensity training pressure is insufficient. This study explored the association between positive parenting style and academic burnout among PETE students, testing the chain mediating roles of physical activity and meaning in life based on Conservation of Resources theory.

**Methods:**

A cross-sectional design was used. In December 2025, a stratified sampling method was applied to survey 1,192 PETE students from 15 provinces in China (age range 18–24 years, *M* = 19.5, *SD* = 1.27; 54.4% male). Participants completed the Short Form of the Egna Minnen Beträffande Uppfostran (S-EMBU) for positive parenting style, the Physical Activity Behavior Scale, the Chinese Meaning in Life Questionnaire (C-MLQ), and the Adolescent Learning Burnout Inventory (ALBI). Chain mediation analysis was performed using the PROCESS macro (Model 6) in SPSS 25.0, and indirect effects were tested with the bootstrap method (5,000 resamples).

**Results:**

Correlation analysis showed significant pairwise correlations among positive parenting style, physical activity, meaning in life, and academic burnout. After controlling for gender and grade, the chain mediation model was supported. Positive parenting style was negatively associated with academic burnout (*β* = −0.110, *p* < 0.001). Three indirect pathways were identified: (1) via physical activity (indirect effect = −0.307, 95% CI [−0.340, −0.274]), (2) via meaning in life (indirect effect = −0.019, 95% CI [−0.030, −0.011]), and (3) via the sequential path of physical activity to meaning in life (indirect effect = −0.048, 95% CI [−0.061, −0.037]).

**Conclusion:**

Positive parenting style was negatively associated with academic burnout among PETE students. The independent mediating effect of physical activity was the strongest (effect = −0.307, 63.3% of total indirect effect). The chain mediation pathway (positive parenting style → physical activity → meaning in life → academic burnout) showed a small effect size (indirect effect = −0.048, contributing only 9.9% of the total indirect effect), indicating it plays a minor, auxiliary role rather than a dominant mechanism. Due to the cross-sectional design, causality cannot be inferred. Future longitudinal studies are needed to confirm these associations.

## 1 Introduction

Academic burnout is a relatively common psychological problem among university students worldwide (Schaufeli et al., 2002), with prevalence rates ranging from 35% to 88% across different regions (Park et al., 2020; Olmos-Bravo et al., 2025; Sun and Liu, 2018; Kaggwa et al., 2021; Mhata et al., 2023). As competition in higher education intensifies, academic burnout among university students, especially those in teacher education and other specific majors, has received increasing attention. Academic burnout not only impairs students’ immediate learning quality and mental health but may also weaken their career preparation and future professional development potential in the long run. Within this general issue, Physical Education Teacher Education (PETE) students are a unique group whose situation deserves particular attention. They need to complete heavy theoretical courses while continuously engaging in high intensity professional skills training. At the same time, they bear the role expectation of future physical education teaching and health promotion. However, existing research on academic burnout has mostly focused on general university students (Chen and Chen, 2025; Osorio Guzman et al., 2020; Loscalzo et al., 2024; Wang and Jiang, 2025a), with insufficient in-depth exploration of the specific risks and protective mechanisms for PETE students. Identifying key factors associated with their academic burnout and analyzing the underlying patterns of association is urgently needed to safeguard this group’s mental health and improve the quality of professional talent cultivation.

Parenting style, particularly positive parenting characterized by emotional warmth and support, has been identified as a distal factor associated with academic outcomes (Padilla-Walker et al., 2021). However, its association with academic burnout among PETE students, and the potential mechanisms involved, remain underexplored.

Drawing on the Conservation of Resources (COR) theory, which posits that individuals strive to acquire and protect valuable resources to cope with stress, this study proposes a sequential pathway from external family resources to individual behavioral resources and then to internal meaning-based psychological resources (Hobfoll, 2011). Specifically, we aim to test whether physical activity and meaning in life serve as sequential mediators in the statistical association between positive parenting style and academic burnout among PETE students.

In summary, this study focuses on PETE students and uses a cross-sectional survey to explore the association between positive parenting style and academic burnout, with a particular emphasis on testing the chain mediating roles of physical activity and meaning in life. The findings are expected to provide theoretical basis and practical insights for constructing a comprehensive psychological support system that integrates health behavior promotion and value cultivation for this group. Given the cross-sectional design, causality cannot be inferred, and all path interpretations are based on statistical associations.

## 2 Literature review and hypothesis development

### 2.1 Positive parenting style and academic burnout

Parenting style is understood as a comprehensive context. It refers to a relatively stable pattern of behavioral tendencies and emotional atmosphere that parents display in the process of raising their children (Darling and Steinberg, 2017). Through daily interactions, emotional expression, rule setting, and expectation transmission, it systematically influences child development. Parenting style is a core element in the family environment that affects individual development. It profoundly shapes children’s cognitive patterns, emotion regulation abilities, and behavioral motivation (Seay et al., 2014). According to Diana Baumrind’s classic theoretical framework, parenting styles are often classified into authoritative, authoritarian, permissive, and neglectful types (Peprah, 2022). Among these, the authoritative style, characterized by high responsiveness (warmth and support) and high demandingness (clear rules and expectations), is considered most beneficial for children’s healthy psychosocial adaptation. This parenting style is associated with better psychological adjustment, academic performance, and adaptive strategies (Fuentes et al., 2022).

Academic burnout is an extension of the burnout concept to the academic domain (Maslach and Jackson, 1981). It refers to a state of physical and emotional exhaustion resulting from prolonged academic stress. Its core features are emotional exhaustion, learning detachment, and reduced sense of achievement (Schaufeli et al., 2002). Many studies have confirmed that positive parenting style is a protective factor against academic burnout (Kim and Kim, 2021; Mikkonen et al., 2023), while negative parenting style is a significant risk factor (Zhu et al., 2023; Yang et al., 2025). Specifically, a family environment filled with emotional warmth, understanding (Kochhar, 2025), and reasonable autonomy support provides individuals with important psychological safety and emotional stability resources (Sbicigo and Dell’Aglio, 2012). This initial family resource is positively associated with children maintaining a positive coping attitude when facing academic challenges and may be linked to lower levels of academic burnout (Lindfors et al., 2018). In contrast, negative parenting styles characterized by excessive interference, harsh punishment, rejection, or emotional coldness may erode children’s self-efficacy and induce or exacerbate emotional exhaustion, detachment, and low achievement in their studies (Ding et al., 2019). Moreover, when parents’ care for their children is contingent on academic performance, it significantly associated with a higher risk of academic burnout. This makes children’s self-worth overly dependent on academic achievement, making them more vulnerable to emotional exhaustion when facing challenges (Lavrijsen et al., 2023). Most existing research has focused on general university students, with little specific investigation into Physical Education Teacher Education (PETE) students, a special group facing both high intensity training and academic pressure.

Therefore, based on the Conservation of Resources theory and existing evidence, we propose Hypothesis H1: Positive parenting style negatively associated with academic burnout.

### 2.2 Positive parenting style, physical activity, and academic burnout

A positive parenting style characterized by warmth and reasonable autonomy support directly satisfies adolescents’ basic psychological needs. This is associated with psychological capital such as hope and resilience, which are key personal resources for coping with academic challenges (Carmona-Halty et al., 2022). Positive parenting style is associated with children’s development of the healthy habit of regular physical activity (Su et al., 2025). Regular physical activity, especially of moderate to vigorous intensity, can positively affect academic performance by improving cognitive function, attention, and classroom behavior (Coe et al., 2006). As an important health behavior, physical activity is voluntary body movement undertaken to maintain or enhance physical fitness. Physical activity is not merely a process of energy expenditure but is also regarded as an effective personal resource building activity. Regular physical activity is associated with enhanced physical and psychological resources (Yeh et al., 2016).

The influence of positive parenting style on physical activity has received substantial research support. A family environment that provides emotional warmth and encourages autonomous exploration satisfies individuals’ basic psychological needs and is associated with higher intrinsic motivation and self-efficacy (Ryan and Deci, 2000). The autonomous motivation and self-efficacy that are associated with such a supportive family environment are also linked to more sustained behavioral participation. This indicates that physical activity becomes internalized from an external requirement into a positive habit and lifestyle (Cachon-Zagalaz et al., 2023). From a developmental perspective, long term exposure to a controlling or cold family atmosphere may weaken an individual’s autonomy and self-regulation ability (Ryan and Deci, 2000). When parents adopt neglectful or laissez faire strategies, children’s physical activity levels tend to be the lowest (Hennessy et al., 2010). Therefore, positive parenting style can be seen as an important social contextual resource that is associated with individuals’ engagement in physical activity.

The statistical association between physical activity and lower academic burnout also has theoretical and empirical foundations. From a resource acquisition perspective, participating in physical activity directly produces multiple benefits. Physiologically, regular physical activity significantly enhances physical function, health related fitness, and overall quality of life (Castro et al., 2023). Psychologically, increasing physical activity effectively relieves stress, improves mood, and enhances perceived control (Paluska and Schwenk, 2000). Regular physical activity is associated with replenishment of personal resources that may be depleted by academic stress, and this pattern is linked to higher self-efficacy and resilience. Such resource-related patterns are also associated with lower emotional exhaustion and, consequently, lower levels of academic burnout (Chen et al., 2022). In addition, the positive emotional experiences gained from physical activity and the social support from team sports are linked to lower feelings of helplessness and detachment in learning (Chen et al., 2022).

Synthesizing the two pathways above, physical activity may play a key mediating role between positive parenting style and academic burnout. The initial psychological resources linked to parental emotional support and encouragement are positively correlated with children’s adoption and persistence in physical activity. The continuous physiological and psychological resources associated with this behavior are, in turn, correlated with better coping with academic stress and maintaining learning engagement, and are associated with lower burnout risk. This mediation pathway reveals the dynamic process through which family environment resources are transmitted to the academic domain via behavioral pathways. Therefore, we propose Hypothesis H2: Physical activity mediates the relationship between positive parenting style and academic burnout.

### 2.3 Positive parenting style, meaning in life, and academic burnout

Meaning in life is a psychological experience in which individuals have a clear sense of purpose and direction in their lives and perceive their own existence as valuable and important (Martela and Steger, 2016). Meaning in life is not only a cognitive understanding but also an emotional commitment and identification (King et al., 2006). Meaning in life is also a core psychological resource associated with maintaining mental health and positive development. When coping with stress and adversity, a strong sense of meaning in life is associated with lower psychological distress, such as anxiety and depression, playing a key protective role (Brassai et al., 2011). Recent cross-cultural evidence further confirms that the experience dimension of meaning in life is positively associated with hope and negatively associated with anxiety and depression among university students, supporting its robust protective function across different cultural contexts (Yıldırım et al., 2021).

Based on the Conservation of Resources theory, individuals strive to acquire, maintain, and protect resources they value. These resources include material, social, and psychological assets (Taylor et al., 2000). Because meaning in life provides a sense of direction, coherence, and purpose, it is regarded as a key psychological resource. Meaning in life is regarded as a key psychological resource. Individuals with higher meaning in life tend to report better stress coping and sustained effort (Wolfram, 2023; Duckworth et al., 2007; Ryan and Deci, 2000). From this theoretical perspective, the family is the primary shaper of an individual’s meaning system. Therefore, parenting style has a foundational influence on the formation of meaning in life.

First, positive parenting style is an important source of meaning in life. One study clearly showed that maternal emotional warmth is associated with fewer internalizing problem behaviors, and this association is statistically mediated by meaning in life (Zhang et al., 2024). A warm, responsive, and autonomy supportive parenting environment satisfies children’s basic psychological needs for care, competence, and autonomy. When the social environment supports these needs, it is associated with higher intrinsic motivation, social development, and well-being (Ryan and Deci, 2000). The satisfaction of these psychological needs is not only a mediating factor for good adjustment but is also directly related to higher levels of subjective wellbeing and meaning in life. For example, research has found that parental autonomy support enhances individuals’ subjective wellbeing through the chain mediation path of basic psychological need satisfaction to meaning in life (Lin et al., 2025).

Second, meaning in life is a key psychological resource associated with lower levels of academic burnout. Students’ levels of learning burnout (including emotional exhaustion, detachment, and low achievement) are significantly negatively correlated with their meaning in life (Arens and Morin, 2016). Meaning in life is not a passive trait but an active resource that shapes cognitive appraisal and emotional experience. When students have a clear sense of meaning and purpose, they are more likely to reappraise academic challenges as part of their personal growth. Studies show that students with higher academic motivation also report higher levels of meaning in life, and this meaning in turn is associated with reduced school burnout (Güngör and Sari, 2022). This process suggests that meaning in life is associated with lower emotional exhaustion and greater learning engagement. Longitudinal studies further reveal the role of meaning in life. Research indicates that meaning in life is directly associated with lower learning burnout, and it is also indirectly associated via psychological resilience (Wang et al., 2022). Psychological resilience, as a coping resource, is associated with better management of academic stress (Yeo and Yap, 2023).

In summary, positive parenting style provides individuals with the emotional security and psychological foundation necessary to construct a positive sense of meaning in life. This is an input of initial social emotional resources. The strong sense of meaning in life thus cultivated, as a higher order internal meaning based psychological resource, directly empowers individuals in the academic domain. It helps students interpret stress and maintain motivation, and is associated with a lower risk of academic burnout. Therefore, meaning in life likely plays a key mediating role between positive parenting style and academic burnout.

Accordingly, we propose the following hypothesis: Meaning in life mediates the relationship between positive parenting style and academic burnout.

### 2.4 Positive parenting style, physical activity, meaning in life, and academic burnout

Synthesizing the analyses of the independent mediation pathways above, this study proposes that physical activity and meaning in life may not simply act in parallel between positive parenting style and academic burnout. Instead, they may form a theoretically logical chain path. Based on the Conservation of Resources theory, the dynamic process of individual resources follows a sequence. Concrete resources are typically associated with more immediate coping responses, while abstract resources show patterns of correlation consistent with longer-term psychological processes (Hobfoll, 1989). Therefore, the association between positive parenting style and academic burnout may occur sequentially through being associated with physical activity first, and then with meaning in life.

For PETE students, this transmission process from concrete behavioral resources (physical activity) to abstract psychological resources (meaning in life) may be particularly salient given their professional context. First, positive parenting style, especially the emotional support it provides, has been confirmed by multiple studies as a key factor associated with regular physical activity habits in children, adolescents, and even young adults (Hennessy et al., 2010). For PETE students, participating in physical activity is not only a general health promoting behavior but also a core component of their professional competence development. This behavior is associated with better emotional state, less stress, higher sense of control, and greater self-confidence (Han et al., 2025). It is also directly linked to the development of their motor skills as professional capital. The abundant vitality, positive emotions, and professional efficacy thus obtained constitute key primary physical and psychological resources. According to the broaden and build theory of positive psychology, positive emotions and self-efficacy broaden immediate cognitive and behavioral resources and subsequently build enduring personal resources, laying the foundation for coping with more complex psychological challenges (Fredrickson, 2001). Extending this logic, evidence suggests that meaning in life functions alongside other protective factors such as resilience in buffering the negative effects of psychological distress on maladaptive behaviors (Çiçek et al., 2025). When individuals are in such a resource rich state, they are more capable and motivated to engage in deep reflection, integration, and value seeking, making it easier to construct and enhance their meaning in life.

Second, physical activity itself may be associated with meaning in life. Physical activity correlates with a sense of meaning through multiple pathways: investing in health, achieving goals, and social connections. These pathways together suggest that physical activity is associated with the construction and enhancement of meaning in life (Zhao et al., 2025; Jiang and Bian, 2025). For PETE students, this experience is more easily integrated with their future professional values. Physical activity becomes not only a form of self-regulation but also a practice of professional identity and mission, thus contributing more directly to the formation of meaning in life (Toubassi et al., 2023).

Therefore, this study tests a chain mediation model for PETE students. Positive parenting style, as an initial social–emotional resource, is statistically associated with individuals’ development and maintenance of physical activity. The physical and psychological resources that co-occur with physical activity are further associated with higher meaning in life. Finally, higher meaning in life is associated with lower levels of academic burnout.

**Figure 1:**
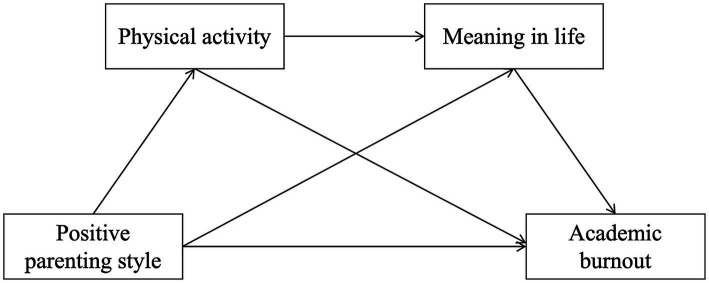
The chain mediation effect model.

Based on the above analysis, we propose Hypothesis H4: Physical activity and meaning in life form a chain mediation pathway between positive parenting style and academic burnout (positive parenting style → physical activity → meaning in life → academic burnout) (see Figure 1. The chain mediation effect model).

## 3 Methodology

### 3.1 Procedures and participants

This study used a cross-sectional design. In December 2025, we selected the sample using stratified sampling. The stratification variables were geographical region (east, central, west, south, and north) and school type (physical education colleges within normal universities, and physical education departments within comprehensive universities). Within each geographical region, we randomly selected three provinces based on the list of higher education institutions published by the Ministry of Education, resulting in a total of 15 provinces. Within each selected province, we randomly chose one university that met the school type criteria. The final sample covered 15 universities across 15 provinces in China. In each selected university, we used cluster sampling to recruit undergraduate students majoring in physical education or sports training from the first to the fourth year, taking classes as clusters. The class advisors distributed the questionnaires. Before distribution, the research team provided specialized training to the physical education teachers, covering key points such as voluntary participation procedures, withdrawal mechanisms, and confidentiality requirements.

Subsequently, the class advisors gave the questionnaires to the physical education major students. Participants completed the questionnaires under the supervision of their class advisors. During this process, the teachers explained the voluntary participation principle, the meaning of the questions, and the right to withdraw. After data collection, we excluded 108 invalid responses based on predefined criteria: (1) abnormally short or long completion time, and (2) inattentive response patterns (e.g., repetitive or contradictory answers).

The final sample included 1,192 participants. Participants were aged 18 to 24 years (mean = 19.5, SD = 1.265). Among them, 648 were male (54.36%) and 544 were female (45.64%). The grade distribution was as follows: 471 first year students (39.51%), 342 s year students (28.69%), 266 third year students (18.96%), and 113 fourth year students (9.48%). For household registration type, 580 participants were from urban areas (48.66%) and 612 from rural areas (51.34%). The effective response rate was 91.69%. Exclusion criteria were: (1) severe physical illness, (2) history of mental illness, and (3) receipt of psychological treatment within the past 3 months.

Data were collected through an online platform^1^ using an electronic questionnaire. The questionnaire consisted of five parts: basic information (grade, gender, height, weight), the Positive Parenting Style Scale (S-EMBU), the Physical Activity Scale (PA), the Meaning in Life Questionnaire (MLQ), and the Academic Burnout Inventory (ALBI). Before data collection, all participants signed an electronic informed consent form. After data collection, all information was anonymized to ensure anonymity. The anonymized dataset will be stored in accordance with institutional ethical guidelines and data management policies, and will be available for research purposes for at least 5 years after publication. Qualified researchers may apply for access to the data but must sign a formal data sharing agreement to ensure participant privacy and comply with the original informed consent terms.

### 3.2 Ethics approval and consent to participate

This study strictly followed the ethical principles of the Declaration of Helsinki. The research protocol was approved by the Ethics Committee of Chengdu Sport University (approval number: CTYLL2025235). Before formal participation, all participants signed an electronic informed consent form through the online platform. The informed consent form detailed the study purpose, voluntary participation principle, confidentiality agreement, and the right to withdraw from the study at any time without any liability. To ensure participant privacy, all data were anonymized during collection and analysis. No personal identifying information was retained in the final dataset.

### 3.3 Measurement tools

#### 3.3.1 Positive parenting style scale

We used the Emotional Warmth subscale of the Short Form of the Egna Minnen Beträffande Uppfostran (S-EMBU) developed by Arrindell et al. (1999) to measure positive parenting style. The full scale includes 21 items for each parent across three dimensions: rejection (6 items, e.g., “My parents often lose their temper with me for no reason”), emotional warmth (7 items, e.g., “My parents often praise me”), and overprotection (8 items, e.g., “I wish my parents would not worry too much about what I am doing”). We used only the Emotional Warmth subscale to measure positive parenting style because this dimension best fits the concept of “positive resource supply” in the Conservation of Resources theory, whereas rejection and overprotection are negative or ambivalent dimensions. Items were rated on a four-point Likert scale (1 = never, 4 = always). Higher scores indicated greater consistency between the described behavior and how the parent treated the participant. The Cronbach’s alpha coefficient for this subscale in the present study was 0.837.

#### 3.3.2 Physical activity scale

We used the Physical Activity Behavior Scale revised by Deqing (1994) to measure participants’ physical activity over the past month. The scale contains three factors: physical activity intensity, physical activity duration, and physical activity frequency. Intensity and frequency were rated on a five-point scale, while duration was rated on a four-point scale. Physical activity volume was calculated using the formula: Activity volume = intensity × duration × frequency. Higher scores indicated greater physical activity volume per session. The Cronbach’s alpha coefficient for this scale in the present study was 0.950.

#### 3.3.3 Meaning in life scale

Meaning in life was measured using the Meaning in Life Questionnaire (MLQ) developed by Steger et al. (2006). The scale contains two dimensions with a total of 10 items: presence of meaning (5 items, e.g., “I understand my life’s meaning very well”) and search for meaning (5 items, e.g., “I am looking for something that makes my life meaningful”). Items were rated on a seven-point Likert scale (1 = completely disagree, 7 = completely agree). Higher scores indicated greater alignment between the item description and the participant’s own feelings. The Cronbach’s alpha coefficient for this scale in the present study was 0.826.

#### 3.3.4 Academic burnout scale

Academic burnout was measured using the Adolescent Learning Burnout Inventory (ALBI) developed by Lian et al. (2005). The scale contains three dimensions with a total of 16 items: physical and mental exhaustion (4 items, e.g., “I have been feeling empty lately and do not know what to do”), academic detachment (5 items, e.g., “I feel that I do not understand anyway, so it does not matter whether I study or not”), and reduced sense of achievement (7 items, e.g., “I can handle exams well”). Items were rated on a five-point Likert scale (1 = very consistent, 5 = very inconsistent). Higher scores indicated that the item description was less consistent with the participant’s own feelings. The Cronbach’s alpha coefficient for this scale in the present study was 0.833.

### 3.4 Data analysis

The data analysis consisted of three steps.

Several procedural measures were implemented to reduce potential common method bias. Participants were assured of the anonymity and confidentiality of their responses in the informed consent form. The academic burnout scale (ALBI) included reverse-scored items (e.g., “I can handle exams well”), which help disrupt mechanical response patterns. The four scales were presented in separate sections with distinct instructions to minimize item carryover effects.

First, we assessed common method bias using Harman’s single factor test. It should be noted that this test is only a statistical control method and cannot completely eliminate the method bias inherent in self-report data. Future research should use multi source reports (e.g., from parents or teachers) or objective measurements (e.g., accelerometers) for cross validation.

Second, we performed descriptive statistics and correlation analysis. For all variables (positive parenting style, physical activity, meaning in life, and academic burnout), we calculated means, standard deviations, and Pearson correlation coefficients.

Third, we conducted chain mediation analysis. We used the PROCESS macro (version 3.4) in SPSS 25.0 to build a chain mediation model, testing the transmission pathways from positive parenting style to academic burnout through physical activity and meaning in life. Gender, grade, and body mass index were included as covariates. We used the bootstrap resampling method (5,000 iterations) with 95% confidence intervals to test the indirect effects.

## 4 Results

### 4.1 Common method bias test

Because the questionnaire data were obtained through self reports from participants, we used Harman’s single factor test to examine potential common method bias. We conducted an exploratory factor analysis on all items of the Positive Parenting Style Scale, Physical Activity Scale, Meaning in Life Questionnaire, and Academic Burnout Inventory. Principal component analysis was used to extract common factors, and we examined the partial correlation after extracting the first common factor. The results showed that four factors had eigenvalues greater than one. The first factor explained 30.362% of the variance, which was below the critical threshold of 40%. This indicated that no significant common method bias was present in this study.

### 4.2 Descriptive statistics and correlations

We performed descriptive statistics and Pearson correlation analyses on the four variables (positive parenting style, physical activity, meaning in life, and academic burnout). The results are shown in Table 1. All variables showed significant intercorrelations.

**Table 1:** Descriptive statistics and correlation analysis of variables.

Variable	*M*	*SD*	Skewness	Kurtosis	Positive parenting style	Physical activity	Meaning in life	Academic burnout
Positive parenting style	22.910	1.380	0.699	0.356	1			
Physical activity	74.930	29.627	−0.722	−0.896	0.450**	1		
Meaning in life	46.000	9.013	−0.150	−0.580	0.391**	0.671**	1	
Academic burnout	49.940	9.600	0.061	−0.824	−0.485**	−0.848**	−0.674**	1

**p < 0.01. Independent samples *t*-tests were conducted to examine gender differences across the four variables. The results showed no significant gender differences for positive parenting style (male: *M* = 22.89, *SD* = 1.39; female: *M* = 22.93, *SD* = 1.36), *t* = −0.49, *p* = 0.624; meaning in life (male: *M* = 45.82, *SD* = 9.08; female: *M* = 46.21, *SD* = 8.93), *t* = −0.74, *p* = 0.458; or academic burnout (male: *M* = 50.08, *SD* = 9.64; female: *M* = 49.77, *SD* = 9.55), *t* = 0.55, *p* = 0.581. Male students reported significantly higher physical activity levels than female students (male: *M* = 77.85, *SD* = 29.12; female: *M* = 71.46, *SD* = 29.84), *t* = 3.71, *p* < 0.001.

Positive parenting style was negatively correlated with academic burnout (*r* = −0.485, *p* < 0.01). Positive parenting style was positively correlated with physical activity (*r* = 0.450, *p* < 0.01) and positively correlated with meaning in life (*r* = 0.391, *p* < 0.01). Physical activity was positively correlated with meaning in life (*r* = 0.671, *p* < 0.01) and negatively correlated with academic burnout (*r* = −0.848, *p* < 0.01). Meaning in life was negatively correlated with academic burnout (*r* = −0.674, *p* < 0.01).

### 4.3 Positive parenting style and academic burnout among university students: chain-mediated effects

To examine the predictive effects of positive parenting style, physical activity, and meaning in life on academic burnout, we conducted hierarchical regression analyses. Positive parenting style, physical activity, and meaning in life were entered as independent variables, and academic burnout was the dependent variable. The results are shown in Table 2.

**Table 2:** Regression analysis of positive parenting style, physical activity, meaning in life, and academic burnout.

Variable	Academic burnout			
	*β*	*T*	*F*	*R* ^2^
Positive parenting style	−0.485	−19.109***	365.160	0.234
Physical activity	−0.848	−55.087***	3034.591	0.718
Meaning in life	−0.674	−31.448***	988.988	0.453

***p < 0.001.

Positive parenting style negatively associated with academic burnout (*β* = −0.485, *t* = −19.109, *p* < 0.001). Physical activity negatively associated with academic burnout (*β* = −0.848, *t* = −55.087, *p* < 0.001). Meaning in life negatively associated with academic burnout (*β* = −0.674, *t* = −31.448, *p* < 0.001).

We then tested the chain mediation model. Positive parenting style (X) was the independent variable, academic burnout (Y) was the dependent variable, and physical activity (W1) and meaning in life (W2) were the mediators. After controlling for demographic variables (gender and grade), we examined the chain mediating effects of physical activity and meaning in life on the relationship between positive parenting style and academic burnout. We used the PROCESS macro (version 3.4) in SPSS 25.0, specifically Model 6, to test the hypotheses. We used 5,000 bootstrap resamples and 95% confidence intervals (CIs) to test the indirect effects.

As shown in Table 3, positive parenting style had a significant negative association with academic burnout (*β* = −0.485, *p* < 0.001; 95% CI [−0.534, −0.435]). This indicated that higher scores on positive parenting style were associated with lower severity of academic burnout, supporting Hypothesis 1.

**Table 3:** Mediation effect values and effect sizes.

Effect path	Effect	BOOT SE	BOOT LLCI	BOOT ULCI	Relative mediation effect
Total effect	−0.485***	0.025	−0.534	−0.435	100%
Direct effect	−0.110***	0.017	−0.142	−0.078	22.680%
Total indirect effect	−0.375***	0.018	−0.410	−0.341	77.320%
Indirect effect 1 (physical activity)	−0.307***	0.017	−0.340	−0.274	63.299%
Indirect effect 2 (meaning in life)	−0.019***	0.005	−0.030	−0.011	3.918%
Indirect effect 3 (physical activity & meaning in life)	−0.048***	0.006	−0.061	−0.037	9.897%

Indirect effect 1: positive parenting style → physical activity → academic burnout; Indirect effect 2: positive parenting style→ meaning in life → academic burnout; Indirect effect 3: positive parenting style → physical activity → meaning in life → academic burnout. ***p < 0.001.

The relationship between positive parenting style and academic burnout was mediated by physical activity (*p* < 0.001; 95% CI [−0.340, −0.274]). This indicated that physical activity played a mediating role in the association between positive parenting style and academic burnout, supporting Hypothesis 2.

Meaning in life also mediated the relationship between positive parenting style and academic burnout (*p* < 0.001; 95% CI [−0.030, −0.011]). This indicated that meaning in life played a mediating role in the association, supporting Hypothesis 3.

**Figure 2:**
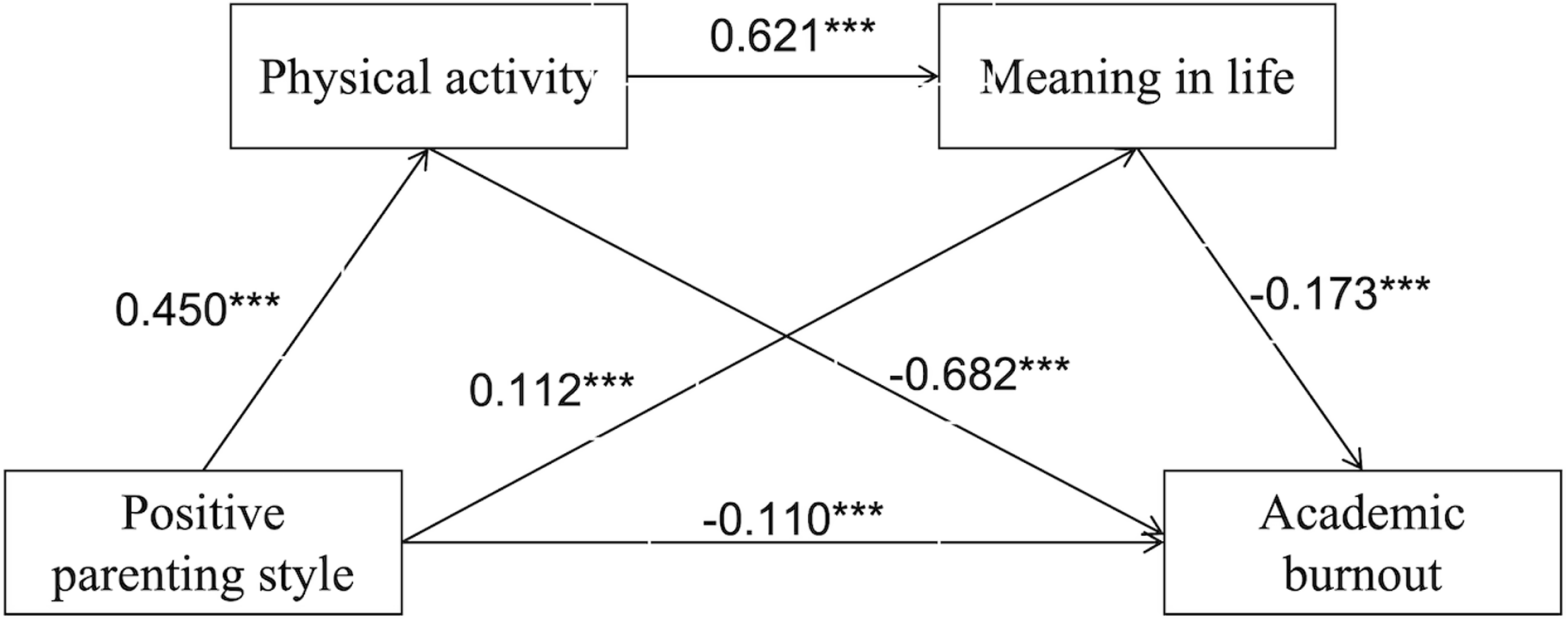
Model of chain mediating roles of physical activity and meaning in life between positive parenting style and academic burnout. ***p < 0.001.

Finally, physical activity and meaning in life sequentially mediated the relationship (*p* < 0.001; 95% CI [−0.061, −0.037]). This supported the sequential path from positive parenting style through physical activity to meaning in life and then to academic burnout, confirming Hypothesis 4. The specific paths are shown in Figure 2.

In addition, the effect sizes of the three indirect paths and the total effect were all statistically significant (*p* < 0.001). The total effect was −0.485. The direct effect was −0.110, accounting for 22.680% of the total effect. The total indirect effect was −0.375, accounting for 77.320% of the total effect. For the indirect paths: Path 1 (positive parenting style → physical activity → academic burnout) had an effect size of −0.307, accounting for 63.299% of the total indirect effect. Path 2 (positive parenting style → meaning in life → academic burnout) had an effect size of −0.019, accounting for 3.918% of the total indirect effect. Path 3 (positive parenting style → physical activity → meaning in life → academic burnout) had an effect size of −0.048, accounting for 9.897% of the total indirect effect.

Furthermore, the multicollinearity test results (shown in Table 4) indicated that all VIF values were less than five, suggesting no multicollinearity issue. The Durbin Watson (D W) value was approximately two, indicating no autocorrelation in the model and that the sample data did not show any interrelationships.

**Table 4:** Linear regression analysis results.

Variable	Unstandardized coefficients		Standardized coefficients	*t*	*p*	Collinearity statistics	
	*B*	Std. Error	Beta			VIF	Tolerance
Constant	92.508	2.473	-	37.412	0.000	-	-
Positive parenting style	−0.765	0.115	−0.110	−6.677	0.000	1.277	0.783
Physical activity	−0.221	0.007	−0.682	−33.366	0.000	1.967	0.508
Meaning in life	−0.185	0.021	−0.173	−8.742	0.000	1.852	0.540
*R* ^2^	0.748						
*R*^2^ adj	0.747						
F	1174.601***						
D-W	2.021						

***p < 0.001.

## 5 Discussion

Based on the Conservation of Resources theory, this study aimed to explore the associations among positive parenting style, physical activity, meaning in life, and academic burnout in Physical Education Teacher Education (PETE) students, as well as the underlying patterns of these associations. Cross sectional data analysis showed that positive parenting style was not only directly negatively associated with academic burnout but also associated with it through the chain mediation pathway of physical activity to meaning in life. This pattern suggests that a positive family environment may be associated with promoting regular exercise behavior, which in turn is associated with a higher level of meaning in life, ultimately relating to a lower level of academic burnout. The identification of this chain path provides an integrative perspective for understanding the potential sequential associations among external resources, concrete behaviors, and abstract psychological cognition.

### 5.1 Direct association of positive parenting style

The results of this study provide more detailed evidence for understanding the association between positive parenting style and academic burnout. First, positive parenting style was associated with lower levels of academic burnout in PETE students. This supports previous findings that a warm family environment, as an adaptive resource, is negatively associated with academic burnout in university students (Molina Moreno et al., 2025; Andrade et al., 2023). For PETE students who bear both the load of skills training and academic pressure, the emotional warmth and understanding provided by parents may not only be general psychological support but may also be associated with the satisfaction of their specific needs for autonomy and competence. This view is highly consistent with the core psychological needs of Self Determination Theory (Deci and Ryan, 2012). Specifically, this supportive family environment helps students maintain intrinsic motivation when facing demanding training schedules and form positive self-beliefs when coping with academic challenges. It may be related to the critical and stable psychological resource base needed to cope with the dual challenges of the professional domain. This finding further confirms that a positive family emotional environment is a key developmental resource. Through mechanisms such as providing emotional buffering and cultivating psychological resilience (Ren et al., 2019; Ong et al., 2006), it shows negative correlations with burnout in students from professionally demanding fields. It also suggests that when supporting such students, attention should be paid to the emotional resources that their family system can provide. Notably, the direct association between positive parenting style and academic burnout was relatively modest (*β* = −0.110). This is consistent with the Conservation of Resources theory, which suggests that distal resources are linked to well-being mainly through more proximal resources (Hobfoll, 2011). Given the cross-sectional design, it is equally possible that lower burnout leads to more positive perceptions of parenting.

### 5.2 Mediating role of physical activity

Beyond the direct effect, this study further revealed the mediating role of physical activity. The data showed that physical activity mediated the relationship between positive parenting style and academic burnout. This is consistent with the theoretical model of family support, health behavior, and mental health (Heidari et al., 2025), and extends this association to the specific domain of academic burnout (Wang and Jiang, 2025b). Notably, for PETE students, this mediating role may be strengthened by their professional context. Their physical activity is not merely leisure or recreation but is systematic training integrated into daily life with clear professional skill development goals. This high level of integration and functional attribute may allow emotional support from the family to be more directly linked to behavioral motivation. At the same time, the psychological benefits linked to training, such as a sense of physical mastery and positive emotions, are correlated with lower emotional exhaustion (Liu et al., 2022). Therefore, the prominence of this pathway in this group not only confirms the general model (Wang and Jiang, 2025b), but also reveals the possible synergistic enhancement effect between professional practice and health behavior on psychological functioning. The physical activity pathway accounted for 63.3% of the total indirect effect, substantially more than the other pathways. This may reflect the unique context of PETE students, for whom physical training is an integral part of daily academic life rather than an optional leisure activity. Previous studies have linked regular physical activity to healthier stress-related physiological profiles and greater self-efficacy (Paluska and Schwenk, 2000; Chen et al., 2022). However, the cross-sectional data cannot determine the temporal order of these variables.

### 5.3 Mediating role of meaning in life

In addition to the behavioral pathway, this study also verified the cognitive emotional pathway, namely the mediating role of meaning in life. This is consistent with extensive research showing that meaning in life, as a key psychological resource, is negatively correlated with various stress indicators (Dulaney et al., 2018; Yousaf Raza et al., 2019). Specifically, this study found that among PETE students, perceived parental emotional warmth was associated with a higher level of meaning in life, and a higher level of meaning in life was associated with a lower level of academic burnout. The independent indirect pathway through meaning in life was small (3.9% of the total indirect effect). This is consistent with cross-cultural evidence showing that meaning in life reliably correlates with lower psychological distress, but its unique statistical contribution may be modest when other correlates are included in the model (Yıldırım et al., 2021). This pattern suggests that meaning in life may function more as a downstream correlate than as a standalone mediator. Given its small effect size, meaning in life alone does not appear to be a primary pathway in the statistical model. The psychological benefits linked to structured professional training—such as a sense of control and belonging—may contribute to the broader resource network, but their unique correlation with lower burnout through meaning in life alone is limited.

### 5.4 Chain mediating role

Most importantly, this study provides an integrative sequential perspective by validating the chain mediation path of physical activity to meaning in life. This echoes the Conservation of Resources theory’s perspectives on resource gain spirals and the sequential associations between concrete and abstract resources (Hobfoll et al., 2018; Cui et al., 2023). Positive parenting style, as an emotional resource, shows positive correlations with regular physical activity (Pugliese and Tinsley, 2007). Physical activity in turn is associated with more direct physical and psychological resources, such as higher levels of vitality and positive emotional states (Shraga and Shirom, 2009) and was associated with meaning in life in our statistical model. For PETE students, the statistical associations among physical practice, professional value identification, and meaning in life are consistent with their professional profile (Sun et al., 2025).

Notably, this chain mediation path was statistically significant but had a small effect size (indirect effect = −0.048, accounting for only 9.9% of the total indirect effect). This indicates that this path plays a minor, auxiliary role in the overall association. The much larger effect of the physical activity-only pathway (63.3%) confirms that, in this cross-sectional model, physical activity itself is the strongest correlate of lower academic burnout, not the additional step through meaning in life. The small effect size may also indicate that the link between physical activity and meaning in life varies across individuals—perhaps stronger among those who actively reflect on how their training connects to their professional values. From a theoretical perspective, validating this sequential path is valuable for revealing possible resource transformation patterns, but its limited effect size warrants caution in deriving practical implications. Longitudinal research is needed to determine whether these variables unfold in the temporal sequence implied by the resource cascade hypothesis.

This sequential model closely links the fields of health behavior research and meaning psychology. It suggests a possible sequential association between behavior change and meaning construction, responding to calls for integrating action and cognitive pathways to more fully understand psychological development (Schwarzer and Gutiérrez-Doña, 2009; Narayanan et al., 2011). If these cross-sectional patterns are confirmed by longitudinal research, it would be valuable to examine whether physical activity and meaning in life show sequential associations over time.

### 5.5 Practical implications and future directions

Based on the patterns found in this study, several directions for future research can be identified. It must be emphasized that the cross-sectional design cannot establish any causal relationships. All practical implications below are preliminary hypotheses requiring longitudinal or experimental validation.

First, the physical activity pathway was the strongest correlate of lower burnout, accounting for 63.3% of the total indirect effect. This suggests that, for PETE students, maintaining regular, structured physical training is a particularly robust marker of lower academic burnout. If confirmed by longitudinal research, this would indicate that understanding the scientific nature of professional training and its positive correlations with mood and energy should be a central focus in research on PETE student well-being. Given the dominant effect size of this pathway, it deserves proportionally greater attention in future investigations.

Second, the significant but small chain mediation pathway (9.9%) provides preliminary cross-sectional evidence consistent with a sequential resource model. Whether combining physical activity with reflection on professional meaning yields benefits beyond physical activity alone is a hypothesis that requires rigorous experimental testing. Given the small effect size, the practical significance of this pathway remains uncertain.

Finally, the modest direct association with positive parenting style (*β* = −0.110) and the small independent pathway through meaning in life (3.9%) suggest that these factors, while statistically significant correlates, play limited roles in the overall model. Future research could examine whether home-school cooperation is associated with higher levels of perceived parental emotional warmth, and whether meaning in life contributes more substantially when examined alongside other protective factors such as resilience (Çiçek et al., 2025).

In summary, these cross-sectional findings highlight physical activity as the primary factor meriting further investigation. The chain mediation and meaning-in-life pathways, though statistically significant, contribute modest additional explanatory power. Future longitudinal and experimental research is essential to determine whether the statistical patterns reported here reflect genuine associations over time.

### 5.6 Limitations and future directions

This study has several limitations that need to be considered when interpreting the results and that point to directions for future research. First, the cross-sectional design cannot infer causal relationships among variables. Although the theoretical model suggests a logical sequence from positive parenting style to physical activity, then to meaning in life, and finally to academic burnout, the possibility of reverse or bidirectional associations also exists. For example, high academic burnout might reduce exercise motivation or lower meaning in life, thereby affecting the perception of positive parenting style. Future research should use longitudinal follow up or experimental intervention designs to examine the temporal order and causal effects among the variables. Intensive longitudinal designs, such as daily diary studies, could examine whether day-to-day changes in physical activity are temporally associated with subsequent changes in meaning in life and burnout.

Second, although stratified sampling was used, only one university per province was selected, which may constrain representativeness. Future studies could sample multiple institutions per region to improve generalizability.

Third, all data came from student self-reports, which may be subject to common method bias. Although statistical tests indicated that this bias was not significant, future research could incorporate multiple sources of data, such as parent reports, objective physical activity monitoring devices (e.g., accelerometers), or teacher evaluations, to improve measurement objectivity and accuracy. Relatedly, although procedural measures and statistical testing were employed, common method bias remains a concern. Harman’s single-factor test is a diagnostic rather than a control technique and can only detect severe cases of method bias. More rigorous control methods—such as the unmeasured latent method factor approach within a confirmatory factor analysis framework, or the inclusion of a marker variable—were not applied. The exclusive reliance on self-report measures may have inflated the observed correlations among variables. Future research should incorporate multi-source data (e.g., parent reports, teacher evaluations, objective physical activity monitoring via accelerometers) and employ more sophisticated statistical controls to mitigate the impact of common method bias.

Fourth, several potentially relevant variables—such as peer support, personality traits, academic performance, and family socioeconomic status (SES)—were not measured and may account for part of the observed associations. In particular, family SES may correlate with positive parenting style, access to physical activity resources, and academic burnout. Failure to control for SES may have confounded the observed associations. Future research should include these variables as covariates to rule out their potential confounding effects.

Fifth, this study focused on PETE students, a specific group with professional distinctiveness. While this enhances the internal depth of the study, it may also limit the direct generalizability of the findings to other university student populations. Future research could test the generalizability of the model across student groups from different majors and explore the potential moderating role of major type.

Sixth, although the chain mediation model is informative, it is not the only possible explanatory model. The relationships among variables may be more complex. For instance, meaning in life might also promote persistence in physical activity. Future research could consider and test other theoretical models, such as those including moderating variables (e.g., gender, social support), or explore other potential mediating variables (e.g., psychological resilience, academic self-efficacy), to more comprehensively understand the complex network among family environment, individual behavior, psychological resources, and academic adaptation. Future studies could also examine additional mediators. For instance, Çiçek found that resilience and meaning in life jointly mediated the link between loneliness and problematic social media use (Çiçek et al., 2025). A similar approach could test whether resilience adds to the chain mediation model reported here.

In summary, this study preliminarily reveals the chain associations among positive parenting style, physical activity, meaning in life, and academic burnout, providing a new integrative perspective for understanding the development of PETE students. Future research can further validate and deepen these findings by improving research designs, expanding samples and measurement methods, and exploring more complex models, thereby providing a more solid foundation for theory and practice.

## 6 Conclusion

Based on the Conservation of Resources theory, this study explored the associations between positive parenting style and academic burnout among PETE students, with a focus on testing the potential chain mediating roles of physical activity and meaning in life.

Cross-sectional data analysis showed that students who perceived more positive parenting style tended to have lower levels of academic burnout. This association was statistically explained by three indirect pathways. The pathway through physical activity alone accounted for the largest proportion (63.3% of the total indirect effect). The chain mediation pathway (positive parenting style → physical activity → meaning in life → academic burnout) showed a small effect size (indirect effect = −0.048, contributing only 9.9%), indicating it plays a minor, auxiliary role. The pathway through meaning in life alone was even smaller (3.9%). Thus, the primary correlate of lower academic burnout in this model is physical activity itself, not the additional step through meaning in life.

These findings provide an integrative perspective for understanding the complex connections among family environment, health behavior, deep cognitive resources, and academic adaptation. They suggest that for PETE students, emotional support from the family may not only be a direct psychological buffer but may also function through being associated with their maintenance of regular exercise behavior, which is core to their profession. The physical and psychological resources accumulated through such exercise behavior are also associated with a clearer sense of meaning in life, which in turn is associated with greater resilience to academic stress.

These findings have two main implications. Theoretically, they provide cross-sectional evidence consistent with a resource cascade: distal family resources may be linked to academic well-being through successive associations with behavioral and then cognitive resources. Practically, the results highlight physical activity as a particularly robust correlate of lower burnout in PETE students. If confirmed by longitudinal research, this would suggest that maintaining structured physical training—and potentially pairing it with reflection on professional meaning—could be a useful focus for future intervention studies. All practical interpretations remain preliminary given the cross-sectional design. However, it must be emphasized that the cross-sectional nature of this study limits causal inference. Future research needs to use longitudinal designs or intervention experiments to verify the temporal sequences and causal directions among these variables. In addition, incorporating objective physical activity measurements, multi-source report data, and testing the generalizability of the model across different student groups will be important next steps.

Despite these limitations, the results of this study still have potential implications. If confirmed by longitudinal research, these patterns would be consistent with a multi-level framework in which family emotional environment, regular exercise, and meaning in life are all correlates of lower burnout. However, given the cross-sectional design, no causal or interventional conclusions can be drawn. These findings serve as hypotheses for future longitudinal and experimental research, not as evidence-based recommendations for practice.

## Data Availability

The original contributions presented in the study are included in the article/Supplementary material, further inquiries can be directed to the corresponding author.

## Footnotes

1
https://www.wenjuan.com/s/UZBZJvYWmq/

## References

[ref1] Andrade D. Ribeiro I. J. S. Prémusz V. Maté O. (2023). Academic burnout, family functionality, perceived social support and coping among graduate students during the Covid-19 pandemic. Int. J. Environ. Res. Public Health 20:4832. doi: 10.3390/ijerph20064832, 36981741 PMC10049259

[ref2] Arens A. K. Morin A. J. S. (2016). Relations between teachers’ emotional exhaustion and students’ educational outcomes. J. Educ. Psychol. 108:800. doi: 10.1037/edu0000105

[ref3] Arrindell W. A. Sanavio E. Aguilar G. Sica C. Hatzichristou C. Eisemann M. . (1999). The development of a short form of the Embu: its appraisal with students in Greece, Guatemala, Hungary and Italy. Pers. Individ. Differ. 27, 613–628. doi: 10.1016/S0191-8869(98)00192-5

[ref4] Brassai L. Piko B. F. Steger M. F. (2011). Meaning in life: is it a protective factor for adolescents’ psychological health? Int. J. Behav. Med. 18, 44–51. doi: 10.1007/s12529-010-9089-6, 20960241

[ref5] Cachon-Zagalaz J. Carrasco-Venturelli H. Sanchez-Zafra M. Zagalaz-Sanchez M. L. (2023). Motivation toward physical activity and healthy habits of adolescents: a systematic review. Children 10:659. doi: 10.3390/children10040659, 37189907 PMC10136410

[ref6] Carmona-Halty M. Salanova M. Schaufeli W. B. (2022). The strengthening starts at home: parent–child relationships, psychological capital, and academic performance–a longitudinal mediation analysis. Curr. Psychol. 41, 3788–3796. doi: 10.1007/s12144-020-00898-8

[ref7] Castro R. L. G. Manalansan L. M. Sibug V. B. (2023). Physical activity engagement across human life cycles: a scoping review. Int. J. Multidiscip. Appl. Bus. Educ. Res 4, 2786–2800. doi: 10.11594/ijmaber.04.08.18, 41611218

[ref8] Chen J. Chen G. (2025). Academic burnout among Chinese college students: a study based on Fsqca method. Acta Psychol. 253:104701. doi: 10.1016/j.actpsy.2025.104701, 39798488

[ref9] Chen K. Liu F. Mou L. Zhao P. Guo L. (2022). How physical exercise impacts academic burnout in college students: the mediating effects of self-efficacy and resilience. Front. Psychol. 13:964169. doi: 10.3389/fpsyg.2022.964169, 36438387 PMC9691659

[ref10] Çiçek İ. Ünsal F. Korkmaz Z. (2025). Loneliness and problematic social media use among university students: exploring the mediating roles of meaning in life and resilience. Psychol. Health Med., 1–15 Online ahead of print. doi: 10.1080/13548506.2025.2581894, 41165093

[ref11] Coe D. P. Pivarnik J. M. Womack C. J. Reeves M. J. Malina R. M. (2006). Effect of physical education and activity levels on academic achievement in children. Med. Sci. Sports Exerc. 38, 1515–1519. doi: 10.1249/01.mss.0000227537.13175.1b, 16888468

[ref12] Cui H. Bi X. Zhou X. Zhang W. Ma Y. (2023). Family function and adolescent altruistic behavior: the multiple mediating effects of self-affirmation and psychological resilience. Front. Psychol. 14:1184985. doi: 10.3389/fpsyg.2023.1184985, 37546475 PMC10400445

[ref13] Darling N. Steinberg L. (2017). “Parenting style as context: an integrative model,” in Interpersonal Development, eds. Laursen B. Zukauskiene R. (London, UK: Routledge).

[ref14] Deci E. L. Ryan R. M. (2012). “Self-determination theory,” in Handbook of Theories of social Psychology, eds. Lange P. A. M. Van Kruglanski A. W. Higgins E. T., Thousand Oaks, California: Sage 416–436.

[ref15] Deqing L. (1994). Stress level of college students and its relationship with physical exercise. Chin. Ment. Health J. 8:2. doi: 10.3321/j.issn:1000-6729.1994.01.020

[ref16] Ding X. Zhibing Z. Ge G. “The study on the relationship among parenting style, negative perfectionism and academic burnout of college students,” in 2019 10th International Conference on Information Technology in Medicine and Education (ITME) Piscataway, New Jersey (2019), 237–240.

[ref17] Duckworth A. L. Peterson C. Matthews M. D. Kelly D. R. (2007). Grit: perseverance and passion for long-term goals. J. Pers. Soc. Psychol. 92, 1087–1101. doi: 10.1037/0022-3514.92.6.1087, 17547490

[ref18] Dulaney E. S. Graupmann V. Grant K. E. Adam E. K. Chen E. (2018). Taking on the stress-depression link: meaning as a resource in adolescence. J. Adolesc. 65, 39–49. doi: 10.1016/j.adolescence.2018.02.011, 29525578 PMC5932235

[ref19] Fredrickson B. L. (2001). The role of positive emotions in positive psychology: the broaden-and-build theory of positive emotions. Am. Psychol. 56, 218–226. doi: 10.1037//0003-066X.56.3.218, 11315248 PMC3122271

[ref20] Fuentes M. C. Garcia O. F. Alcaide M. Garcia-Ros R. Garcia F. (2022). Analyzing when parental warmth but without parental strictness leads to more adolescent empathy and self-concept: evidence from Spanish homes. Front. Psychol. 13:1060821. doi: 10.3389/fpsyg.2022.1060821, 36544447 PMC9760939

[ref21] Güngör A. Sari H. I. (2022). Effects of academic motivation on school burnout in Turkish college students. Int. J. Adv. Couns. 44, 414–431. doi: 10.1007/s10447-022-09477-x, 35791378 PMC9247920

[ref22] Han X. Li H. Niu L. (2025). How does physical education influence university students’ psychological health? An analysis from the dual perspectives of social support and exercise behavior. Front. Psychol. 16:1457165. doi: 10.3389/fpsyg.2025.1457165, 40040663 PMC11877448

[ref23] Heidari Z. Kianimoghadam A. S. Arani A. M. Bakhtiari M. (2025). Investigating the relationship between loneliness, physical activity, and internet addiction: the mediating role of academic burnout and self-control. Iran. J. Psychiatry 20, 330–344. doi: 10.18502/ijps.v20i3.19040, 41185675 PMC12579799

[ref24] Hennessy E. Hughes S. O. Goldberg J. P. Hyatt R. R. Economos C. D. (2010). Parent-child interactions and objectively measured child physical activity: a cross-sectional study. Int. J. Behav. Nutr. Phys. Act. 7:71. doi: 10.1186/1479-5868-7-71, 20929570 PMC2964559

[ref25] Hobfoll S. E. (1989). Conservation of resources: a new attempt at conceptualizing stress. Am. Psychol. 44, 513–524. doi: 10.1037/0003-066X.44.3.513, 2648906

[ref26] Hobfoll S. E. (2011). “Conservation of resources theory: its implication for stress, health, and resilience,” in The Oxford Handbook of Stress, Health, and Coping, ed. Folkman S. (New York: Oxford University Press), 127–147.

[ref27] Hobfoll S. E. Halbesleben J. Neveu J.-P. Westman M. (2018). Conservation of resources in the organizational context: the reality of resources and their consequences. Annu. Rev. Organ. Psychol. Organ. Behav. 5, 103–128. doi: 10.1146/annurev-orgpsych-032117-104640

[ref28] Jiang Y. Bian T. (2025). The effects of physical exercise on college students’ pro-social behavior: the chain mediating role of sense of meaning in life and subjective well-being. Front. Psychol. 16:1604700. doi: 10.3389/fpsyg.2025.1604700, 40606900 PMC12213650

[ref29] Kaggwa M. M. Kajjimu J. Sserunkuma J. Najjuka S. M. Atim L. M. Olum R. . (2021). Prevalence of burnout among university students in low-and middle-income countries: a systematic review and meta-analysis. PLoS One 16:e0256402. doi: 10.1371/journal.pone.0256402, 34460837 PMC8405021

[ref30] Kim J. Y. Kim E. (2021). Effect of positive parenting styles as perceived by middle school students on academic achievement and the mediation effect of self-esteem and academic engagement. Sustainability 13:13233. doi: 10.3390/su132313233

[ref31] King L. A. Hicks J. A. Krull J. L. Del Gaiso A. K. (2006). Positive affect and the experience of meaning in life. J. Pers. Soc. Psychol. 90:179. doi: 10.1037/0022-3514.90.1.179, 16448317

[ref32] Kochhar R. I. (2025). Psychological safety and emotional regulation in families. Int. J. Indian Psychol. 13, 2682–2694. doi: 10.25215/1303.245

[ref33] Lavrijsen J. Soenens B. Vansteenkiste M. Verschueren K. (2023). When insecure self-worth drains students’ energy: academic contingent self-esteem and parents’ and teachers’ perceived conditional regard as predictors of school burnout. J. Youth Adolesc. 52, 810–825. doi: 10.1007/s10964-023-01749-y, 36807227

[ref34] Lian R. Yang L. X. Wu L. H. (2005). Relationship between professional commitment and learning burnout of undergraduates and scales developing. Acta Psychol. Sin. 37, 632–636.

[ref35] Lin T. Jian L. Liang H. Xiong F. Zhang L. (2025). The relationship between parental autonomy support and subjective well-being: chain mediating effects of basic psychological needs and sense of meaning in life. Psychol. Health Med. 1-21, 1–21. doi: 10.1080/13548506.2025.2581899, 41187945

[ref36] Lindfors P. Minkkinen J. Rimpelä A. Hotulainen R. (2018). Family and school social capital, school burnout and academic achievement: a multilevel longitudinal analysis among Finnish pupils. Int. J. Adolesc. Youth 23, 368–381. doi: 10.1080/02673843.2017.1389758

[ref37] Liu J. Gao S. Zhang L. (2022) Effects of physical exercises on emotion regulation: a meta-analysis. Medrxiv 2022-07 doi: 10.1101/2022.07.04.22277120

[ref38] Loscalzo Y. Rice K. G. Giannini M. (2024). Psychometric properties of the Italian Oldenburg burnout inventory (student version) and measurement invariance with the USA. Curr. Psychol. 43, 8241–8251. doi: 10.1007/s12144-023-05020-2

[ref39] Martela F. Steger M. F. (2016). The three meanings of meaning in life: distinguishing coherence, purpose, and significance. J. Posit. Psychol. 11, 531–545. doi: 10.1080/17439760.2015.1137623

[ref40] Maslach C. Jackson S. E. (1981). The measurement of experienced burnout. J. Organ. Behav. 2, 99–113. doi: 10.1002/job.4030020205

[ref41] Mhata N. T. Ntlantsana V. Tomita A. M. Mwambene K. Saloojee S. (2023). Prevalence of depression, anxiety and burnout in medical students at the University of Namibia. S. Afr. J. Psychiatry 29:2044. doi: 10.4102/sajpsychiatry.v29i0.2044, 37292521 PMC10244924

[ref42] Mikkonen K. Veikkola H.-R. Sorkkila M. Aunola K. (2023). Parenting styles of Finnish parents and their associations with parental burnout. Curr. Psychol. 42, 21412–21423. doi: 10.1007/s12144-022-03223-7

[ref43] Molina Moreno P. Fernández Gea S. Pérez-Fuentes M. D. C. Molero Jurado M. D. M. Gázquez Linares J. J. (2025). Family functionality as a mediator in the relationship between humanization and academic burnout in adolescents. Front. Psychol. 15:1520912. doi: 10.3389/fpsyg.2024.1520912, 39881708 PMC11774834

[ref44] Narayanan V. K. Zane L. J. Kemmerer B. (2011). The cognitive perspective in strategy: an integrative review. J. Manage. 37, 305–351. doi: 10.1177/0149206310383986

[ref45] Olmos-Bravo Z. M. Sánchez-Ortí J. V. Grevet E. H. Balanzá-Martínez V. (2025). Prevalence and associated factors of burnout among health sciences students in Spain: a systematic review. Trends Psychiatry Psychother. 47:e20240805. doi: 10.47626/2237-6089-2024-0805, 38833324 PMC12904265

[ref46] Ong A. D. Bergeman C. S. Bisconti T. L. Wallace K. A. (2006). Psychological resilience, positive emotions, and successful adaptation to stress in later life. J. Pers. Soc. Psychol. 91, 730–749. doi: 10.1037/0022-3514.91.4.730, 17014296

[ref47] Osorio Guzman M. Prado Romero C. Parrello S. Bazan Riveron G. E. (2020). Psychometric characteristics and factor structure of the school burnout inventory student (Sbi-U-9) in Mexican university students. Rev. Iberoam. Diagn. Eval.-E Aval. Psicol. 2, 141–150. doi: 10.21865/Ridep55.2.10

[ref48] Padilla-Walker L. M. Son D. Nelson L. J. (2021). Profiles of helicopter parenting, parental warmth, and psychological control during emerging adulthood. Emerg. Adulthood 9, 132–144. doi: 10.1177/2167696818823626

[ref49] Paluska S. A. Schwenk T. L. (2000). Physical activity and mental health: current concepts. Sports Med. 29, 167–180. doi: 10.2165/00007256-200029030-00003, 10739267

[ref50] Park S. Y. Andalibi N. Zou Y. Ambulkar S. Huh-Yoo J. (2020). Understanding students’ mental well-being challenges on a university campus: interview study. JMIR Form Res 4:e15962. doi: 10.2196/15962, 32134393 PMC7082737

[ref51] Peprah E. O. (2022). The parenting style that yields better academic performance in tertiary students. Can. J. Educ. Soc. Stud. 2, 57–72. doi: 10.53103/cjess.v2i1.21

[ref52] Pugliese J. Tinsley B. (2007). Parental socialization of child and adolescent physical activity: a meta-analysis. J. Fam. Psychol. 21, 331–343. doi: 10.1037/0893-3200.21.3.331, 17874918

[ref53] Ren X. Wang Y. Hu X. Yang J. (2019). Social support buffers acute psychological stress in individuals with high interdependent self-construal. Acta Psychol. Sin. 51:497. doi: 10.3724/Sp.J.1041.2019.00497

[ref54] Ryan R. M. Deci E. L. (2000). Self-determination theory and the facilitation of intrinsic motivation, social development, and well-being. Am. Psychol. 55, 68–78. doi: 10.1037//0003-066X.55.1.68, 11392867

[ref55] Sbicigo J. B. Dell’aglio D. D. (2012). Family environment and psychological adaptation in adolescents. Psicologia: Reflexão e Crítica 25, 615–622. doi: 10.1590/S0102-79722012000300022, 41099703

[ref56] Schaufeli W. B. Martinez I. M. Pinto A. M. Salanova M. Bakker A. B. (2002). Burnout and engagement in university students: a cross-national study. J. Cross-Cult. Psychol. 33, 464–481. doi: 10.1177/0022022102033005003

[ref57] Schwarzer R. Gutiérrez-Doña B. (2009). Modelando el cambio en el comportamiento de salud: Cómo predecir y modificar la adopción y el mantenimiento de comportamientos de salud. Rev. Costarric. Psicol. 28, 11–39.

[ref58] Seay A. Freysteinson W. M. Mcfarlane J. (2014). Positive parenting. Nurs. Forum 49, 200–208. doi: 10.1111/nuf.12093, 24898152

[ref59] Shraga O. Shirom A. (2009). The construct validity of vigor and its antecedents: a qualitative study. Hum. Relat. 62, 271–291. doi: 10.1177/0018726708100360

[ref60] Steger M. F. Frazier P. Oishi S. Kaler M. (2006). The meaning in life questionnaire: assessing the presence of and search for meaning in life. J. Couns. Psychol. 53:80. doi: 10.1037/0022-0167.53.1.80

[ref61] Su Z. Yang D. Wang C. Xiao Z. Cai S. (2025). Structural assessment of family and educational influences on student health behaviours: insights from a public health perspective. PLoS One 20:e0333086. doi: 10.1371/journal.pone.0333086, 40996979 PMC12463285

[ref62] Sun H. Z. Liu X. W. (2018). Meta-analysis of factors affecting Chinese university students’ learning burnout. J. Beijing Univ. Aero. Astro. 31:13766:10. doi: 10.13766/j.bhsk.1008-2204.2016.0172

[ref63] Sun P. Ma K. Xu X. Yan L. (2025). How self-efficacy shapes professional identity: the mediating role of meaning in life and self-esteem in pre-service physical education teachers. BMC Psychol. 13:387. doi: 10.1186/s40359-025-02679-z, 40234999 PMC12001478

[ref64] Taylor S. E. Kemeny M. E. Reed G. M. Bower J. E. Gruenewald T. L. (2000). Psychological resources, positive illusions, and health. Am. Psychol. 55, 99–109. doi: 10.1037/0003-066X.55.1.99, 11392870

[ref65] Toubassi D. Schenker C. Roberts M. Forte M. (2023). Professional identity formation: linking meaning to well-being. Adv. Health Sci. Educ. 28, 305–318. doi: 10.1007/s10459-022-10146-2, 35913664 PMC9341156

[ref66] Wang T. Jiang Y. (2025a). The relationship between professional identity and academic burnout among college students majoring in physical education a chain-mediated effect. Front. Psychol. 16:1618909. doi: 10.3389/fpsyg.2025.1618909, 41030344 PMC12477245

[ref67] Wang X. Jiang Y. (2025b). The effect of family support on junior high school students' engagement in physical education—a moderated chain mediation model. Front. Psychol. 16:1606642. doi: 10.3389/fpsyg.2025.1606642, 40831488 PMC12358406

[ref68] Wang Q. Sun W. Wu H. (2022). Associations between academic burnout, resilience and life satisfaction among medical students: a three-wave longitudinal study. BMC Med. Educ. 22:248. doi: 10.1186/s12909-022-03326-6, 35382810 PMC8980514

[ref69] Wolfram H.-J. (2023). Meaning in life, life role importance, life strain, and life satisfaction. Curr. Psychol. 42, 29905–29917. doi: 10.1007/s12144-022-04031-9

[ref70] Yang L. Shrestha S. Zhan S. Cheng F. (2025). Parents' education anxiety and adolescents' academic burnout: the role of parenting style and academic self-efficacy. Pers. Individ. Differ. 238:113082. doi: 10.1016/j.paid.2025.113082

[ref71] Yeh H.-P. Stone J. A. Churchill S. M. Wheat J. S. Brymer E. Davids K. (2016). Physical, psychological and emotional benefits of green physical activity: an ecological dynamics perspective. Sports Med. 46, 947–953. doi: 10.1007/s40279-015-0374-z, 26330207

[ref72] Yeo K.-J. Yap C.-K. (2023). Helping undergraduate students cope with stress: the role of psychosocial resources as resilience factors. Soc. Sci. J. 60, 120–142. doi: 10.1080/03623319.2020.1728501

[ref73] Yıldırım M. Arslan G. Green Z. A. Ashraf F. Sugawara D. Tanhan A. . (2021). Validation and utility of the meaning in life measure for Turkish university students. J. Happiness Health. 1, 40–48. doi: 10.47602/johah.v1i1.2

[ref74] Yousaf Raza A. N. Bhatti Z. G. Mehmood H. J. J. P. Doi M. H. C. (2019). Meaning in life as a moderator of stress in undergraduate students. J. Psychol. Ment. Health Care. 10:8892:2637. doi: 10.31579/2637-8892/062

[ref75] Zhang L. Wang R. Li Y. Chen L. (2024). The impact of maternal emotional warmth on adolescents’ internalizing problem behaviors: the roles of meaning in life and friendship conflict. Front. Psychol. 15:1478610. doi: 10.3389/fpsyg.2024.1478610, 39679149 PMC11638585

[ref76] Zhao H. Li Y. Shi L. (2025). A chain mediation model reveals the association between physical exercise and sense of meaning in life in college students. Sci. Rep. 15:28345. doi: 10.1038/s41598-025-13710-z, 40759714 PMC12322190

[ref77] Zhu Q. Cheong Y. Wang C. Tong J. (2023). The impact of maternal and paternal parenting styles and parental involvement on Chinese adolescents’ academic engagement and burnout. Curr. Psychol. 42, 2827–2840. doi: 10.1007/s12144-021-01611-z

